# Is There a Risk of Recurrence That Would Discourage Sympathicotomy for Hyperhidrosis in Minors?

**DOI:** 10.3390/jcm14228194

**Published:** 2025-11-19

**Authors:** Sara Degiovanni, Francesco Petrella, Ugo Cioffi, Angelo Guttadauro, Francesca Spinelli, Sara Lo Torto, Andrea Cara, Lidia Libretti, Emanuele Pirondini, Antonio Tuoro, Enrico Mario Cassina, Giuseppe Nicolosi, Federico Raveglia

**Affiliations:** 1 Department of Thoracic Surgery, Fondazione IRCCS San Gerardo dei Tintori, 20900 Monza, Italy; francesco.petrella@irccs-sangerardo.it (F.P.); francesca.spinelli@unimi.it (F.S.); sara.lotorto@hotmail.it (S.L.T.); andrea.cara@irccs-sangerardo.it (A.C.); lidia.libretti@irccs-sangerardo.it (L.L.); emanuele.pirondini@irccs-sangerardo.it (E.P.); antonio.tuoro@irccs-sangerardo.it (A.T.); enricomario.cassina@irccs-sangerardo.it (E.M.C.); federico.raveglia@irccs-sangerardo.it (F.R.); 2 Department of Surgery, University of Milan, 20122 Milan, Italy; ugocioffi5@gmail.com; 3 Department of Medicine and Surgery, University of Milano Bicocca, 20158 Milan, Italy; angelo.guttadauro@unimib.it; 4 Department of Thoracic Surgery, University of Milan, 20122 Milan, Italy; giuseppe.nicolosi@unimi.it

**Keywords:** hyperhidrosis, sympathicotomy, sympathectomy, children, recurrence, compensatory sweating

## Abstract

**Background/Objectives**: Primary palmar hyperhidrosis (PPH) is a socially debilitating condition that often begins in adolescence. Although sympathicotomy is a low-risk procedure, there are conflicting opinions about the optimal time for surgery: some recommend it at onset in adolescence, while others are cautious because of the risk of recurrence associated with juvenile neuroplasticity. The primary objective was to assess the recurrence rate; secondary objectives included the management of hyperhidrosis, compensatory sweating onset, and satisfaction. **Methods**: This retrospective cohort study included sympathicotomy procedures for palmar hyperhidrosis performed between 2004 and 2024 in patients younger than 18 years with a preoperative Hyperhidrosis Disease Severity Scale (HDSS) class of 4. Quantitative data are presented as medians and interquartile ranges, while categorical data are presented as numbers and percentages. **Results**: 28 patients were included, of whom 4 underwent single-stage surgery and 24 two-stage surgery. The median age was 17.30 years, with no gender predominance. At a median follow-up of 75 months, the recurrence rate was 10.71%. Twenty-two patients experienced compensatory sweating, with the majority (60.7%) reporting mild symptoms and the remainder reporting moderate. The median patient satisfaction score was 9.34 (range 9–10). **Conclusions**: Even though PPH typically begins during adolescence, there is no consensus on the appropriateness of sympathicotomy for younger patients, primarily due to concerns about recurrence. Our data, characterized by long-term follow-up and large numbers of minors, are consistent with those observed in adults’ cohorts in terms of hyperhidrosis management, compensatory sweating rates, and, particularly, recurrence rates, supporting the surgical approach even at a young age.

## 1. Introduction

Hyperhidrosis is a medical condition characterized by excessive and abnormal sweating, which can affect various regions of the body, even in the absence of stressors or elevated temperatures that would justify its presence. It is a disorder that affects approximately 3% of the global population [1]; in 40% of cases, it is limited to the palmar region, and less frequently, it may also involve axillae and feet.

There is no gender predilection, and, in most cases, the condition manifests from adolescence or even childhood [2].

Hyperhidrosis is further divided into two forms: primary (PHH) and secondary (SHH). In the primary form, it is an isolated condition not associated with underlying systemic disorders. The latter, on the other hand, is associated with endocrinological, infectious, neurological, and psychiatric, as well as neoplastic, cardiovascular, dermatological, and immunological diseases [3].

Primary hyperhidrosis (PHH), although considered an entirely benign condition, causes significant social and psychological issues for the patient. Even though it is not a life-threatening condition, it can have a severe impact on quality of life, especially during adolescence, a time when social interactions are crucial for emotional and psychological development, resulting in embarrassment, social withdrawal, and a significant decline in self-esteem [4]. In particular, hyperhidrosis among children and adolescents is a common cause of depression and anxiety disorders, with percentages that are, respectively, around 40.9% and 31.8% [5].

There are various therapeutic strategies, ranging from less invasive treatments such as the application of topical products and iontophoresis to the administration of botulinum toxin in the affected areas. However, all these treatments are limited by their temporary efficacy [6]. As a result, many patients rely on surgical intervention to achieve a long-lasting outcome.

**Figure 1:**
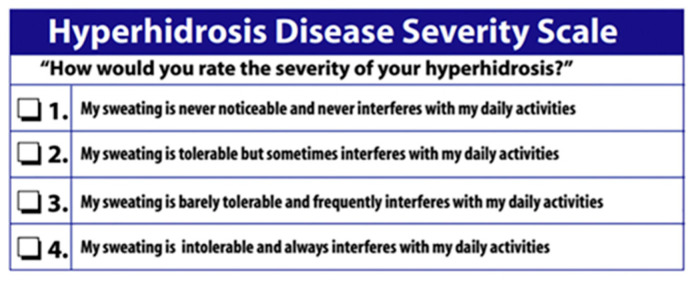
Hyperhidrosis Disease Severity Scale.

Surgery is reserved for the most severe cases of hyperhidrosis, particularly those with a grade 4 on the Hyperhidrosis Disease Severity Scale (HDSS) (Figure 1). Different surgical options are available; they range from simple sectioning of the sympathetic nerve chain (sympathicotomy) to the resection of a nerve segment (sympathectomy). In recent years, there has been increasing interest in the sole sectioning of the nerve fibers exiting the sympathetic chain (ramicotomy).

Currently available evidence demonstrates excellent outcomes in adult patients undergoing this procedure. However, its application in individuals younger than 18 years remains controversial, as it is sometimes discouraged due to concerns that neuroplasticity and Schwann cell–mediated regeneration may predispose to a higher risk of recurrence in this age group [7].

For this very reason, the primary endpoint of our study was to evaluate the recurrence rate of palmar sweating in minors. Furthermore, the management of hyperhidrosis, the incidence and characteristics of compensatory sweating onset, and the overall degree of satisfaction were evaluated as secondary outcomes.

## 2. Materials and Methods

In this retrospective cohort study, we analyzed the surgical interventions of sympathicotomy performed at our center from 2004 to 2024. The following criteria were applied:

- Inclusion criteria: subjects aged <18 years affected by palmar/axillary hyperhidrosis; patients undergoing sympathicotomy; individuals with a preoperative HDSS score of 4; surgical interruption of the sympathetic chain at the R3–R4 level, according to the Consensus Expert [8].
- Exclusion criteria: subjects aged 18 or older; patients reporting excessive sweating in any body area other than the palms or axillae; patients with bradycardia (resting heart rate <55 beats per minute); Body Mass Index (BMI) > 28.

Patients who presented with excessive sweating in other body areas, including the plantar region, were excluded from our study in order to avoid confounding factors, particularly those affecting postoperative satisfaction.

Although all patients included in the study are minors, at our center the procedure is offered only to those who can understand and accept the surgery together with their parents; as a result, most patients fall within the 16 to under 18 years age range.

The analyzed data included the demographic characteristics of the sample and surgical details (surgical time, side, level of sympathetic chain interruption, surgical technique, and approach); peri- and postoperative complications; and surgical outcomes at follow-up.

In particular, patients were evaluated preoperatively to assess the level of perceived palmar/axillary hyperhidrosis using a questionnaire, which allowed the assignment of a value on the HDSS. During the preliminary visit, patients and parents were made aware of possible complications associated with surgery and the risk of developing compensatory sweating. At follow-up, participants were assessed either through in-person outpatient evaluations or telemedicine consultations to document their level of satisfaction, the possible recurrence of palmar hyperhidrosis, and the occurrence, anatomical distribution, and severity of compensatory sweating. At this stage, written informed consent for study participation was obtained from all patients or from their parents or legal guardians in the case of minors.

### 2.1. Surgical Techniques

Surgical procedures at our center are performed with the patient being placed in the lateral decubitus position, contralateral to the side undergoing intervention. The lower leg is bent, and the upper leg is kept extended; the arms are arranged as follows: the lower arm is extended, while the upper one is either hung or folded near the patient’s head. Two thoracoscopic accesses of about one centimeter each are made, the first at the level of the axillary sulcus (usually in the third intercostal space on the anterior axillary line), the other along the inframammary fold at the fourth intercostal space. A 10 mm scope is then inserted into the pleural cavity, and the sympathetic chain is identified and isolated. The sympathetic chain is either blocked (using a 5 mm double arm clip), cut, or cauterized using electrocautery above the upper rib margin (R level), in particular at the level of R3-R4, to treat palmar or palmar/axillary hyperhidrosis. The surgical decision to interrupt the sympathetic chain either with a clip or with electrocautery was made preoperatively by the surgeon together with the patient and the parents. In particular, the choice was based on the fact that sectioning the nerve with electrocautery is permanent, whereas the clip, in case of overly evident side effects, could be removed. The cauterization along the second rib pleural surface is then extended laterally for two centimeters in order to transect Kuntz’s nerves. These accessory nerve fibers are usually responsible for poor surgical results since they reach the brachial plexus without passing through the sympathetic chain. At the end of the surgery a chest drainage (usually 16 Ch) is placed through the inferior thoracoscopic access. The chest tube is either removed at the end of surgery by applying aspiration, or it is maintained until the first postoperative day. In cases of bilateral intervention in the same operating session, after the first side, the patient is turned to the contralateral side to proceed to the second one.

### 2.2. Statistical Analysis

Quantitative data are presented as medians with interquartile ranges (IQRs), while categorical data are expressed as numbers and percentages (%).

## 3. Results

From January 2004 to December 2024, 28 patients with palmar/axillary hyperhidrosis and an HDSS score of 4 were operated on at our center. Of these, 24 patients underwent bilateral sympathicotomy, while 4 underwent unilateral surgery as minors.

The median age recorded was 17.30 years (IQR: 16.44–17.58), with no gender predominance (M:F = 1:1). The demographic and preoperative characteristics are presented in Table 1.

In 20 years we performed 32 unilateral procedures (19 right-sided sympathicotomies and 13 procedures on the left side). The mean operative time was 23.8 min. Pleural drains were removed under suction at the conclusion of the procedure in 20 sides, while in the remaining 12 unilateral cases, they were left in place until the first postoperative day. No intra- or postoperative complications occurred; in particular, no cases required repositioning of a chest drain or conversion to thoracotomy.

At follow-up (median 75 months (IQRs: 59.3–100.9)), the recurrence rate recorded was 10.71% (Table 2); in particular, 7.14% of the subjects reported recurrence of palmar hyperhidrosis but classified it as a grade 2 on the HDSS, and only 1 patient (3.57%) reported a slightly higher grade recurrence (HDSS 3). The onset of recurrence of palmar sweating, although of a lower grade than preoperative HDSS, was reported approximately 1 to 1.5 years after the surgery.

With regard to compensatory sweating (Table 3), 22 patients (78.6%) reported postoperative hyperhidrosis predominantly involving the back, buttocks, and thighs. Nevertheless, the majority (60.7%) described it as mild, manageable, and markedly less bothersome than the symptoms experienced prior to surgery.

Finally, patients were asked to rate their overall satisfaction on a scale from 0 to 10, considering the outcome achieved in the palmar/axillary areas at follow-up, peri- and postoperative pain, and any occurrence of compensatory sweating. The analysis revealed a very high level of general satisfaction, with a mean score of 9.34 (range 9–10).

## 4. Discussion

Primary hyperhidrosis is a rare condition, especially in the adolescent (1.6%) and pediatric (0.6%) populations [9]. Nevertheless, when it occurs in a severe form, it has an important negative impact on patients’ lives, since it affects the subject’s daily activities but especially his social interactions. This is even more important when the condition occurs at a young age because it influences the social sphere in a delicate moment such as adolescence. This is why these patients complain of social distress and anxiety, isolation, and depression. With the advancement of technology and the ability to easily obtain information through the internet, patients increasingly require a medical visit to expose their problem and search for solutions.

Despite a poorly understood etiology of this condition, many treatments are available at the moment. The less invasive options, such as anti-perspirants and oral anti-cholinergic drugs, are easily accessible, even at a young age, but they are rarely able to effectively control the symptoms [10]. Alternatives such as iontophoresis and botulin toxin injection are more invasive and more functional but always temporary. For this reason, patients demand long-lasting solutions to the problem [11].

Definitive options are surgical, such as sympathectomy and sympathicotomy; in particular thoracoscopic sympathicotomy—a surgical intervention that interrupts the sympathetic chain, typically at the R3–R4 level for palmar hyperhidrosis—has proven to be highly effective for managing this condition in the adult population, with efficacy rates reported to be >90%.

Nevertheless, the decision to perform surgery in younger patients remains controversial for several reasons. First, these individuals are minors, and surgical consent must be obtained from their parents or legal guardians. Second, the condition is benign and primarily cosmetic, and therefore generally does not warrant urgent intervention. For this reason, many centers choose to postpone the intervention until the patient reaches adulthood [6]. In addition, although the risk of complications is minimal, it remains a surgical procedure, and, as such, potential risks must be taken into account.

Most studies evaluating surgical outcomes in pediatric patients have demonstrated excellent efficacy, with results comparable to those reported in adults [12,13,14]. Nonetheless, the available evidence is heterogeneous, as a small number of studies have documented higher recurrence rates, occasionally requiring reintervention, following sympathectomy or sympathicotomy in this population.

The majority of studies expressing caution regarding surgery in younger patients report an increased risk of recurrence of palmar hyperhidrosis, particularly in individuals under 16 years of age, due to nerve regeneration or sprouting [7], in most cases, within a year of surgery [15]. Their main concern lies in the neuroplasticity and regenerative potential of the nervous system, which could lead to a higher risk of relapse after surgery. In particular, a retrospective study conducted by Verhaegh et al. [6] reported a 50% recurrence rate in children (under the age of 16) who underwent R3 sympathicotomy, requiring a re-intervention in almost 36% of the cases. However, this is a study that enrolled only 14 patients, all aged less than or equal to 16 years, and despite this, the reported satisfaction rate is still high (7.5 with a range between 4 and 9). Earlier, Law and Ellis [16] reported a higher risk of recurrence in children while providing a longer follow-up (up to 11 years, with a mean follow-up of 6 years); in particular, 38% of the subjects reported mild recurrent sweating of the hands, especially during extreme heat and anxiety. However, patients were still satisfied with their surgical outcomes.

Despite persistent controversies regarding the potential risk of recurrence, most studies in pediatric patients favor the employment of sympathicotomy, particularly due to the consistently high rates of postoperative satisfaction and the clear improvement in quality of life observed. A review of the available literature highlights how recurrence occurs only in a minority of subjects undergoing surgical treatment [17] and that, if it occurs, it generally manifests itself in a milder and more controllable form than the preoperative condition.

Our retrospective analysis demonstrates that, despite a recurrence rate of 3 patients (10.71%), high postoperative satisfaction supports the consideration of surgical intervention in minors with severe palmar hyperhidrosis (HDSS 4), enabling early and meaningful improvements in quality of life and psychosocial well-being. As patient-perceived recurrence was lower than preoperative HDSS scores, no reinterventions were required in this cohort.

### Strengths and Limitations

The strengths of this study consist of the long follow-up period, homogeneity of the data investigated, and the defined age between 16 and 18 years. The limitations of the study include the small number of enrolled patients, the absence of a control group, the non-homogeneous nerve block technique, the retrospective design, and the impossibility of ignoring the subjectivity of the outcomes.

## 5. Conclusions

Palmar hyperhidrosis is a benign condition but can significantly impair the quality of life of subjects, particularly when this occurs at a young age, as it can severely influence social functioning and everyday activities [4]. Thoracoscopic sympathicotomy represents a valid option for both adults and minors suffering from severe palmar hyperhidrosis since the benefits—in terms of symptom control, improved social functioning, and patient satisfaction—clearly outweigh the manageable risks.

Our findings, reporting no increase in recurrence rates, support the view that patients aged 16–18 years who are candidates for sympathicotomy for palmar or palmar/axillary hyperhidrosis should not be excluded from this treatment option.

## Figures and Tables

**Table 1:** HDSS: Hyperhidrosis Disease Severity Scale.

Population	28 Patients
**Sex**	
Male, n (%)	14 (50%)
Female, n (%)	14 (50%)
**Age**, median (IQR)	17.30 (16.44–17.58)
**Preoperative HDSS**	
HDSS grade 4, n (%)	28 (100%)

**Table 2:** HDSS: Hyperhidrosis Disease Severity Scale.

Postoperative Palmar Recurrence According to HDSS (Requiring No Re-Intervention)	
HDSS grade 2, n (%)	2 (7.14%)
HDSS grade 3, n (%)	1 (3.57%)
**Overall palmar recurrence (requiring no re-intervention)**, n (%)	3 (10.71%)
**Satisfaction score at FU**, median on a scale of 1 to 10 (IQR)	9.34/10 (9; 10)

**Table 3:** HDSS: Hyperhidrosis Disease Severity Scale; CS: Compensatory Sweating; N/Ap: Not Applicable; FU: Follow-Up.

Postoperative Compensatory Sweating	
None, n (%)	6 (21.42%)
Mild, n (%)	17 (60.71%)
Moderate, n (%)	5 (17.87%)
Severe, n (%)	0 (0%)
**CS distribution**	
None, n (%)	6 (21.42%)
Back, n (%)	11 (39.28%)
Glutes, n (%)	3 (14.28%)
Back and glutes, n (%)	8 (28.57%)
**Variation in CS from surgery to FU**	
N/Ap, n (%)	6 (21.42%)
No variations in CS, n (%)	17 (60.71%)
Increase in CS, n (%)	5 (17.87%)

## Data Availability

The original contributions presented in this study are included in the article. Further inquiries can be directed to the corresponding author.

## Abbreviations

The following abbreviations are used in this manuscript:

HDSS	Hyperhidrosis Disease Severity Scale
CS	Compensatory Sweating
FU	Follow-Up
VATS	Video Assisted Thoracic Surgery
PHH	Primary Hyperhidrosis
SHH	Secondary Hyperhidrosis
PPH	Primary Palmar Hyperhidrosis
